# Butein Promotes Lineage Commitment of Bone Marrow-Derived Stem Cells into Osteoblasts via Modulating ERK1/2 Signaling Pathways

**DOI:** 10.3390/molecules25081885

**Published:** 2020-04-18

**Authors:** Basem M. Abdallah, Enas M. Ali

**Affiliations:** 1 Department of Biological Sciences, College of Science, King Faisal University, P.O Box 380, Al-Ahsa 31982, Saudi Arabia; eabdelkader@kfu.edu.sa; 2 Department of Botany and Microbiology, Faculty of Science, Cairo University, Cairo 12613, Egypt

**Keywords:** butein, mesenchymal stem cells, BMSCs, osteoblast, adipocyte, ERK1/2

## Abstract

Butein is a phytochemical that belongs to the chalcone family of flavonoids and has antitumor, anti-inflammatory, and anti-osteoclastic bone resorption activities. This study aims to investigate the effects of butein on the differentiation potential of mouse primary bone marrow-derived mesenchymal stem cells (mBMSCs) into osteoblast and adipocyte lineages. Primary cultures of mBMSCs are treated with different doses of butein during its differentiation. Osteoblast differentiation is assessed by alkaline phosphatase (ALP) activity quantification and Alizarin red staining for matrix mineralization, while adipogenesis is assessed by quantification of lipid accumulation using Oil Red O staining. Osteoblastic and adipocytic gene expression markers are determined by quantitative real-time PCR (qPCR). Western blot analysis is used to study the activation of extracellular signal-regulated kinase (ERK1/2). Interestingly, butein promotes the lineage commitment of mBMSCs into osteoblasts, while suppressing their differentiation into adipocytes in a dose-dependent manner. A similar effect of butein is confirmed in human (h) primary BMSCs. Occurring at the molecular level, butein significantly upregulates the mRNA expression of osteoblast-related genes, while downregulating the expression of adipocyte-related genes. The mechanism of butein-induced osteogenesis is found to be mediated by activating the ERK1/2 signaling pathway. To conclude, we identify butein as a novel nutraceutical compound with an osteo-anabolic activity to promote the lineage commitment of BMSCs into osteoblast versus adipocyte. Thus, butein can be a plausible therapeutic drug for enhancing bone formation in osteoporotic patients.

## 1. Introduction

Bone integrity is retained by a dynamic process of bone remodeling, which maintains the balance between bone formation by osteoblasts and bone resorption by osteoclasts. Osteoblasts are resultant from bone marrow-derived mesenchymal stem cells (BMSCs), while osteoclasts are derived from hematopoietic stem cells in bone marrow [1,2]. An excess bone resorption rate and/or dysregulation of bone formation results in osteoporosis [3,4,5]. To date, the treatment for osteoporosis is mainly based on anti-resorptive drugs that target osteoclast-mediated bone resorption such as bisphosphonates, calcitonin, estrogen and Denosumab, a humanized monoclonal antibody against the receptor-activator of nuclear factor kappa-b ligand (RANKL) [6,7]. Conversely, there are few existing anabolic bone therapeutics for osteoporosis including, parathyroid hormone (PTH) and, recently, Romosozumab, a humanized-neutralizing monoclonal antibody against sclerostin, an antagonist of Wnt signaling secreted by an osteocyte [8,9,10,11]. Accordingly, there is a need for developing new anabolic bone drugs with safe processes to directly target the stimulation of bone formation in bone-loss related diseases.

Osteoblast differentiation of BMSCs involves sequential stages of lineage commitment, proliferation, extracellular matrix maturation, and mineralization [12]. Several signaling pathways have been reported to be crucial for mediating the commitment and differentiation of BMSCs into osteoblasts, including Bone morphogenetic protein (BMP), Wnt signal, transforming growth factor beta (TGF-β), adenosine monophosphate (AMP)-activated protein kinase (AMPK), and mitogen-activated protein kinases (MAPKs) [13,14,15]. The MAPK family consists of three major subfamilies: the extracellular signal-regulated kinases (ERKs), the p38 kinases and the Jun, N-terminal kinases (JNKs), that regulate a variety of cellular programs such as cell proliferation, differentiation, and survival [16]. Moreover, ERK is identified as a crucial signal for controlling the lineage commitment of BMSCs into osteoblast or adipocyte, osteoblast proliferation, apoptosis and differentiation by regulating the expression of *Runx2*, ALP activity and cell cycle regulators [17,18,19]. Additionally, the stimulatory effect of anabolic bone factors, such as platelet-derived growth factor (PDGF), and insulin-like growth factor-I (IGF-I) on osteoblastogenesis is mediated by the ERK signaling pathway [20,21]. Therefore, identifying compounds that target the osteogenic differentiation signal of BMSCs, such as the ERK pathway, could provide a pharmacologic approach for developing a new anabolic bone drug. 

Butein (3,4,2′,4′-tetrahydroxychalcone) is a phytochemical product belonging to the chaloconoid, which is a subclass of the flavonoids family. Butein is a major compound in *Toxicodendron vernicifluum*, *Caragana jubata* and *Rhus verniciflua Stokes* [22,23,24]. In vitro and in vivo studies reveal the biological activity of butein as an anti-oxidant [25], anti-fibrogenic [26], anti-inflammatory [27], anti-cancer [28,29] and anti-adipogenic [30] compound. Therefore, butein shows a high potential for the treatment of inflammatory diseases, cancer, and metabolic disorders (including obesity and diabetes) in many preclinical studies [27,31,32,33]. Based on its process, butein exerts anticancer activity by regulating ERK signaling. Butein is demonstrated to inhibit the proliferation of breast cancer cells and the migration and invasion by bladder cancer cells and hepatocarcinoma cells via the modulating ERK signaling pathway [34,35,36], for example.

Interestingly, butein has been reported to inhibit receptor activators of nuclear factor-kappaB (NF-κB) ligand (RANKL)-induced osteoclastogenesis [37] and to reduce the progression of osteoarthritis in rodents [38]. However, despite this potential therapeutic effect of butein for bone loss prevention, no studies have been published on the effect of butein on the differentiation of BMSCs into the osteoblastic cell lineage. We hypothesize that butein may regulate osteogenesis via modulating ERK signaling. Thus, we aim to investigate the effects of butein on mBMSC differentiation into osteoblasts and adipocytes, and to study the role of MAPK/ERK signaling pathways in mediating the effects of butein on osteogenesis. Our data identify butein as a new nutraceutical compound that promotes osteogenesis via activating ERK1/2 signaling.

## 2. Results

### 2.1. Butein Induces Osteoblast Differentiation of mBMSCs

**Figure 1:**
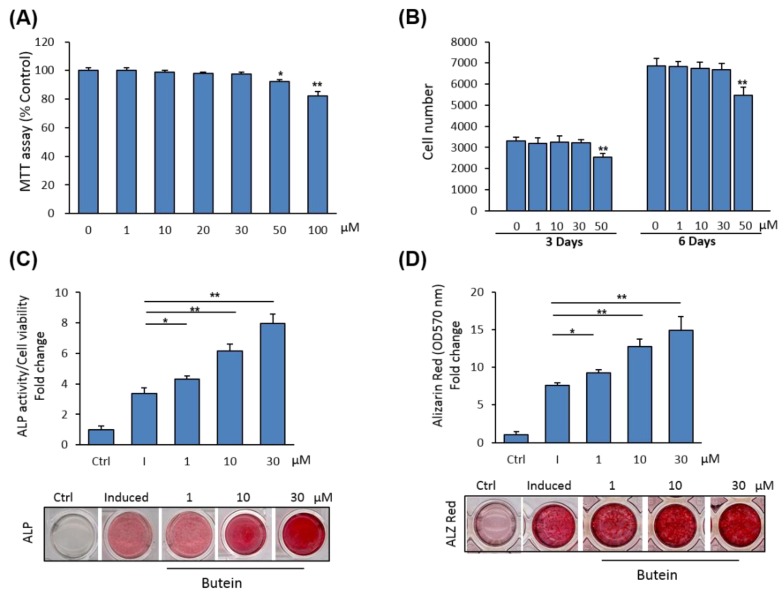
Butein promotes osteoblast differentiation of mBMSCs. (**A**) Cytotoxicity of butein on primary cultures of mBMSCs. Cell viability was determined by an MTT assay. (**B**) The effect of butein at different concentrations on the cell proliferation of BMSCs, as measured by counting cell numbers after 3 and 6 days of treatment. Cells were either non-treated (0) or treated with different concentrations of butein. (**C**) Dose-dependent stimulatory effect of butein on osteogenesis of mBMSCs measured by quantification of ALP activity and (**D**) matrix mineralization with Alizarin red staining after 6 and 12 days of induction, respectively. Cells were either non-induced (Ctrl, control), or induced with an osteogenic cocktail in the absence (I + DMSO) or the presence of different concentrations of butein. Values were represented as fold changes over the control non-induced cells. Stained images were shown. Values are mean ± SD of three independent experiments, (* *p* <0.05, ** *p* <0.005 compared to differentiated cells without butein).

We examined the cytotoxicity of butein on primary cultures of mBMSCs. The effect of different concentrations of butein on cell viability of mBMSCs was measured using an MTT assay. Shown in Figure 1A, butein displayed toxicity on cell viability at concentrations above 30 µM. Similarly, a cell proliferation assay revealed the inhibitory effect of butein on the cell proliferation of mBMSCs at concentrations above 30 µM, as assessed by counting cell numbers after 3 and 6 days in culture. Thus, we used butein at concentrations between 1–30 µM throughout this study (Figure 1B). 

We examined the effect of butein on the differentiation of mBMSCs into osteoblasts. Butein exerts a stimulatory effect on the osteogenesis of mBMSCs in a dose-dependent manner, as demonstrated by increased levels of ALP activity (Figure 1C) and Alizarin Red staining for matrix mineralization (Figure 1D). 

### 2.2. Butein Upregulates the mRNA Expression of Osteoblast-Related Genes in mBMSCs

**Figure 2:**
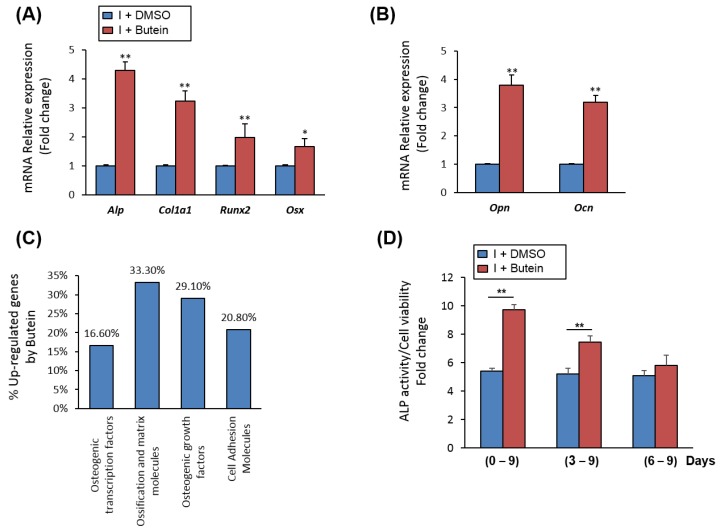
Butein stimulates the upregulation of osteoblast-related gene expression. (**A**) Butein upregulated the gene expression of early and (**B**) late osteogenic markers in mBMSCs during osteogenesis. Cells were induced to osteoblast differentiation for 12 days in the absence (I + DMSO) or the presence of butein (I + butein, 30 µM). Expression of each target gene was normalization to the reference genes and represented as a fold change over differentiated cells without butein. (**C**) Upregulated osteoblast-related genes by butein in mBMSCs as measured by qPCR-based osteogenic gene array analysis. Upregulated genes by butein were categorized according to their osteogenic functions. (**D**) Treatment of mBMSCs with butein at different stages during osteoblast differentiation. Cells were induced to differentiate into osteoblasts and then treated with DMSO (I + DMSO) or butein (30 µM) at day1, day3 and day6 of osteogenesis. ALP activity staining was performed after 9 days for all treatments. Values are mean ± SD of three independent experiments, (* *p* < 0.05, ** *p* < 0.005 compared to differentiated cells without butein).

Treatment of mBMSCs with butein during an osteoblast differentiation course showed significant upregulation early (*Runx2*, *Alp*, *Col1a1*, *Osx)* and late (*Ocn* and *Opn*) of osteoblastic markers by (≥2 fold, *p* < 0.005), as compared to control induced cells with DMSO) (Figure 2A,B). Further, qPCR-based osteogenic gene array analysis revealed significant up-regulation of the gene expression of ossification and matrix-related genes (≥2 fold, *p* < 0.05) by butein in mBMSCs (Figure 2C, Table 1). 

### 2.3. Butein Promotes Osteogenesis of mBMSCs at Early Commitment Stage

To get insight into the mechanism underlying the stimulatory effect of butein on osteogenesis, we examined the effect of butein on mBMSCs at different stages during osteoblast-lineage commitment. Shown in Figure 2D, treatment of mBMSCs with butein during osteogenesis at early (days 0–9) and middle (days 3–9) stages showed a significant increase in ALP activity levels, while late treatment of mBMSCs with butein (days 6–9) was not effective in increasing ALP activity. 

Cells were induced to differentiate into osteoblast without (DMSO) or with 30 µM butein for 6 days. A mouse osteogenesis RT^2^ Profiler™ PCR array was performed for each cDNA sample using the SYBR^®^ Green quantitative PCR method. Up-regulated genes by butein (≥2 fold, *p* < 0.05) in mBMSCs were represented as a fold change over differentiated cells without butein after normalization with reference genes.

### 2.4. Butein Suppresses the Differentiation of mBMSCs into Adipocytes 

**Figure 3:**
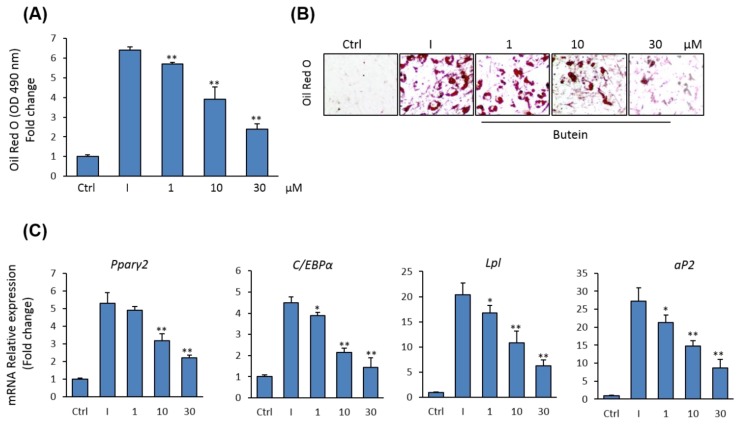
Inhibitory effect of butein on adipogenesis of mBMSCs. (**A**) Dose-dependent inhibitory effect of butein on adipocyte differentiation of mBMSCs. Cells were either non-induced (Ctrl, control), or induced with an adipogenic cocktail in the presence of DMSO (I + DMSO) or different concentrations of butein for 12 days. Lipid accumulation was measured by quantification of Oil Red O staining. (**B**) Images for Oil Red O staining. (**C**) qPCR analysis of mRNA expression of some adipocyte-related genes at day 12 of adipogenesis of mBMSCs in the presence of different concentrations of butein. Each target gene was normalized to the reference genes and represented as a fold change over differentiated cells without butein. Values are mean ± SD of three independent experiments, (* *p* < 0.05, ** *p* < 0.005).

Since osteoblasts and adipocytes in bone marrow are originated from the same precursor BMSCs [39], we examined the effect of butein on adipocyte differentiation of mBMSCs. Butein was shown to inhibit the commitment of mBMSCs into adipocytic cell lineage in a dose-dependent manner, as assessed by Oil Red O quantification and staining for lipid accumulation (Figure 3A,B). Further, butein down-regulated the mRNA expression of both early (*Pparγ2* and *C/ebpα*) and late (*aP2*, and *Lpl*) adipogenic markers in mBMSCs, as measured by qPCR analysis (Figure 3C).

### 2.5. Butein Activates ERK1/2 Signaling Pathway during Osteogenesis.

**Figure 4:**
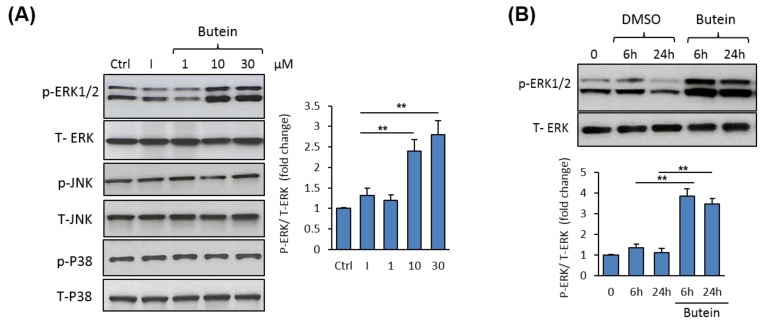
Butein-induced osteogenesis is mediated by activation of ERK1/2 phosphorylation. (**A**) Western blot analysis of MAPK protein expression (Total and phospho-activating extracellular signal-regulated kinase, T-ERK2 and p-ERK1/2; Total and phospho-Jun N-terminal kinase, T-JNK and p-JNK; and total and phospho-P38, T-P38 and p-P38), in mBMSCs treated with different concentrations of butein (1–30 µM). Cells were induced with an osteogenic cocktail (as described in the Materials and Methods) in the absence of non-induced or in the presence of butein for 2 h. (**B**) Western blot analysis of the activation of ERK1/2 phosphorylation by butein in mBMSCs during osteogenesis. Cells were induced with an osteogenic cocktail in the absence or the presence of butein (30 µM) at 6 h and 24 h. Intensity for pERK1/2 was normalized to total ERK and represented as a fold change over control cells. Values are mean ± SD of three independent experiments, (** *p* < 0.005 compared to cells without butein).

To understand the mechanism mediating the stimulatory effect of butein on osteogenesis, we examined the possible regulation of mitogen-activated protein kinases (MAPKs) by butein. Western blot analysis of MAPK expression revealed a dose-dependent stimulatory effect of butein on ERK1/2 phosphorylation without affecting the expression of either p38 or JNK protein phosphorylation (Figure 4A). Further, butein significantly stimulated ERK1/2 phosphorylation during osteoblast differentiation of mBMSCs, as assessed by Western blot analysis (Figure 4B).

### 2.6. Butein Stimulates mBMSCs Differentiation into Osteoblast Versus Adipocyte in ERK1/2-Dependent Pathway 

**Figure 5:**
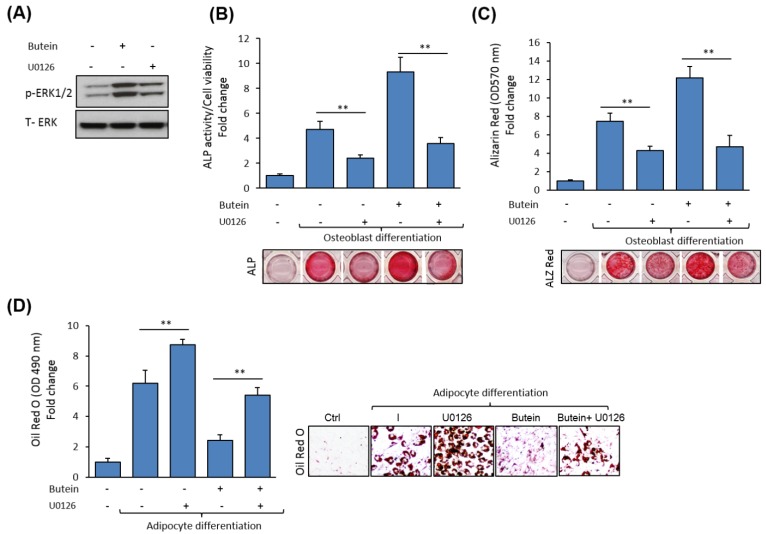
Butein exerts its effect on mBMSCs differentiation via an ERK1/2-dependent pathway. (**A**) Inhibitory effect of the specific ERK1/2 (activating extracellular signal-regulated kinase), U0126 on butein-induced ERK1/2 phosphorylation, as assessed by Western blot analysis. (**B**) The stimulatory effect of butein on osteogenesis in mBMSCs is abolished by blocking ERK1/2 activation, as measured by ALP activity and (**C**) Alizarin Red staining quantification. Cells induced to differentiate into osteoblast were pre-treated with U0126 (10 µM) in the absence or the presence of butein (30 µM) for 6 days (for ALP activity staining) or 12 days (for Alizarin Red staining). (**D**) The inhibitory effect of butein on adipogenesis in mBMSCs is rescued by U0126 pretreatment. Quantification of Oil Red O staining was performed after 12 days. Values are mean ± SD of three independent experiments, (** *p* < 0.005).

To ensure the involvement of ERK1/2 in mediating the effects of butein on mBMSC differentiation, we examined the effect of the blocking of ERK1/2 signaling on osteoblast and adipocyte differentiation of mBMSCs in the presence of butein. The ERK1/2-specific inhibitor, U0126, significantly inhibited ERK1/2 phosphorylation (Figure 5A). Shown in Figure 5B,C, the butein-induced osteogenesis in mBMSCs was significantly abolished by U0126 treatment, as assessed by inhibiting levels of ALP activity and Alizarin Red staining (for matrix mineralization) by 59.3% and 65.4%, respectively, compared to non-treated cells with U0126 (Figure 5B,C). Conversely, pre-treatment of mBMSCs with U0126 rescued the inhibitory effect of butein on the adipogenesis of mBMSCs, as shown by a significantly increased level of lipid accumulation in cells treated with butein compared to non-treated cells (Figure 5D).

### 2.7. Butein Induces the Differentiation of Human (h) BMSCs into Osteoblasts

**Figure 6:**
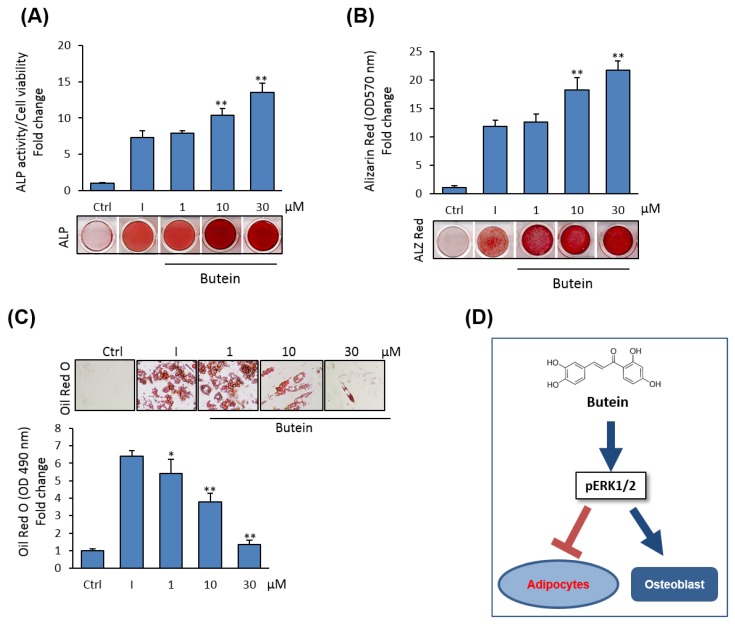
Butein stimulates osteogenesis and inhibits adipogenesis in human (h) BMSCs. (**A**) Dose-dependent stimulatory effect of butein on osteoblast differentiation of hBMSCs as measured by quantification of ALP activity and (**B**) quantification using Alizarin Red staining for matrix mineralization. Images for staining were presented. (**C**) Dose-dependent inhibitory effect of butein on adipocyte differentiation of hBMSCs as assessed by Oil Red O staining quantification. Staining images were presented. (**D**) Schematic diagram demonstrates the stimulatory effect (arrow) of butein on osteogenesis and the inhibitory effect (blunted arrow) of butein on adipogenesis of BMSCs via activating ERK1/2 signaling. Values are mean ± SD of three independent experiments, (* *p* < 0.05, ** *p* < 0.005 compared to differentiated cells without butein).

We further examined the effects of butein on osteoblast and adipocyte differentiation of hBMSCs. Interestingly, butein dose-dependently induced the differentiation of hBMSCs into osteoblasts, while suppressing their differentiation into adipocytes, as assessed by significantly increased ALP activity levels and Alizarin Red staining for osteogenesis, (Figure 6A,B) and reduced levels of Oil Red O staining for adipogenesis (Figure 6C). 

## 3. Discussion

Dietary nutraceuticals are highly attractive therapeutic compounds for the treatment of acute and chronic disorders of human diseases due to their effectiveness, low toxicity, and reduced side effects. Here, we demonstrate that the nutraceutical compound, butein, can effectively promote the early commitment of murine and human BMSCs into osteoblasts, while suppressing their differentiation into adipocytes. The regulatory effect of butein on BMSCs differentiation is mediated by an ERK1/2-dependent signaling pathway.

Butein is a multi-targeted flavonoid with a potential therapeutic effect against several chronic diseases including cancers, inflammatory diseases, obesity, and diabetes [33,40]. Butein displays an anti-cancer effect by stimulating an anti-proliferative effect via, for example, inducing cell cycle arrest at the G2/M phase or by stimulating a pro-apoptotic effect via activation of mitochondria-dependent caspase-3 [41,42]. Several studies report the anti-inflammatory activity of butein and its capacity to inhibit inflammatory reactions via suppression of NF-κB, stimulation of heme oxygenase-1, inhibition of iNOS-derived NO production, and downregulation of IL-6, IL-1β, interferon (IFN)-γ and MMP-9 expression [24,27,43]. Butein is found to exert anti-adipogenic effects and improve glucose tolerance in diet-induced obese and leptin-deficient mice models via the inhibition of central IκB kinase β (IKKβ)/nuclear factor-κB (NF-κB) pathways [44]. Furthermore, butein reduces hyperglycemia-induced diabetic complications in rodents [45,46] and inhibits fibrosis in carbon tetrachloride (CCl4)-induced liver fibrosis in rats [47]. 

Our data identify butein as a novel stimulator of osteoblast differentiation of BMSCs. Previous studies demonstrate the potential therapeutic activity of butein for some bone-related diseases. Butein has been reported to inhibit tumor cell (including myeloma cells) -induced osteoclastogenesis [37] and to reduce the progression of cartilage degradation in a mouse osteoarthritis model [38], for example.

Osteoblast differentiation is initiated by the activation of the master transcriptional regulator, Runt-related transcription factor 2 (*Runx2/Cbfa1*), which regulates the expression of specific genes required for osteoblast maturation and function, including alkaline phosphatase (ALP), type I collagen, osteocalcin, bone sialoprotein, and osteopontin [48,49]. Consistently, our data show the stimulatory effect of butein on upregulating the expression of *Runx2* and its downstream targets. 

The result of BMSCs into osteoblasts or adipocytes is reported to be regulated by the MAPK/ERK signaling pathway [13,48,50,51]. Based on this, we demonstrate that the stimulatory effect of butein on the differentiation of mBMSCs into osteoblasts versus adipocytes is mediated via the activating ERK1/2 signaling pathway. Similarly, increasing ERK1/2 phosphorylation is found to be involved in mediating the differentiation result of BMSCs toward osteoblasts or adipocytes [52,53,54,55]. Additionally, cell shape-dependent control of the lineage commitment of BMSCs through RhoA/ROCK signaling is associated with the ERK/MAPK activation [56]. 

Our results demonstrate the inhibitory effect of butein on adipogenesis of BMSCs. Consistently, butein is reported to inhibit adipocyte differentiation, reduce fat mass and enhance browning of white adipose tissue [57,58,59]. Further, we show that the inhibitory effect of butein on adipogenesis is mediated via ERK1/2-dependent signaling. Other signaling molecules are reported to mediate the inhibitory effect of butein on adipogenesis. These include the activation of p38 mitogen-activated protein kinase/nuclear factor erythroid 2-related factor 2 pathways in pre-adipocyte 3T3-L1 cell lines (p38 MAPK/Nrf2 pathway) [57] and the stimulation of the transforming growth factor-β pathway, followed by STAT3 signaling in the C3H10T1/2 cell line [30]. Thus, the effect of butein on the modulating signaling pathway is cell type-dependent. 

Butein is found to possess estrogenic activity with a high binding affinity to estrogen receptors (ERs) [60]. Interestingly, phytoestrogens that exert estrogen-like biological effects are shown to stimulate osteogenesis and prevent bone loss via ERK1/2 signal pathways. The two phytoestrogens, Genistein and Icariin, induce osteogenesis of osteoprogenitor cells and BMSCs, respectively, via the activation of the ERK1/2 signal pathways [61,62], for example. Additionally, Puerarin, which possesses an estrogen-like structure, suppresses osteoblast apoptosis in an ERK-dependent manner [63]. Thus, it is plausible that the stimulatory effect of butein on osteogenesis via ERK signaling involves the contribution of ERs. However, this hypothetical model needs further experimental work.

Interestingly, our data confirm the stimulatory effect of butein on osteogenesis of mBMSCs in human primary BMSCs, suggesting the plausible potential use of butein as a therapeutic drug for enhancing bone formation in osteoporotic patients. However, pre-clinical studies are needed to prove the osteo-anabolic effect of butein in the bone-loss animal model. 

## 4. Materials and Methods

### 4.1. Cell Cultures and Reagents

Primary male C57BL/6J mouse BMSCs were isolated from 8-weeks-old as previously described [64]. Cells were cultured in an RPMI-1640 medium supplemented with 12% FBS (Thermo Fisher Scientific GmbH, Dreieich, Germany), 12 μM L-glutamine (Thermo Fisher Scientific GmbH, Dreieich, Germany) and 1% penicillin/streptomycin (P/S) (Thermo Fisher Scientific GmbH, Dreieich, Germany). After 24 h, non-adherent cells were removed and cultured in 60 cm^2^. The medium was changed every 3–4 days and cells were washed and regularly sub-cultured.

Human BMSC cells (hBMSCs) were purchased from Cell Applications Inc. (San Diego, CA, USA). Cells were cultured in Dulbecco’s modified Eagle medium (DMEM)/low glucose (Sigma-Aldrich GmbH, Hamburg, Germany) containing 10% FBS (Thermo Fisher Scientific GmbH, Dreieich, Germany) and 1% penicillin/streptomycin according to the manufacturer’s instruction. The medium was changed every 2–3 days. 

Butein (Cat. No. B178) and U0126 (Cat. No. U120) were purchased from (Sigma-Aldrich, Hamburg, Germany).

### 4.2. Cell Toxicity Assay

The cell toxicity of butein was determined by measuring cell viability using an MTT cell proliferation assay kit (Sigma-Aldrich, Hamburg, Germany) according to the manufacturer’s instruction kit. Cells were incubated with an MTT solution to metabolize to formazan, and absorbance was measured at a wavelength of 550  nm for the MTT assay. Values were represented as a fold change for control non-treated cells.

### 4.3. Cell Proliferation Study

The effect of butein on mBMSC proliferation was determined by culturing the cells at 2000 cells/well in 4 well plates. Cells were counted after 3 and 6 days using a hemocytometer. We measured 4–6 biological replicates for each concentration at each time point.

### 4.4. Osteoblast Differentiation

Osteoblast differentiation was stimulated in mBMSCs using an osteogenic induction medium (OIM), which consisted of α-minimum essential medium (α-MEM; Thermo Fisher Scientific GmbH, Dreieich, Germany) supplemented with 10% FBS, 10 mM β-glycerol-phosphate, 100 U/mL of penicillin, 100 mg/mL of streptomycin (Sigma-Aldrich, Hamburg, Germany), and 50 mg/mL of vitamin C (Sigma-Aldrich, Hamburg, Germany). The medium was changed every third day during osteogenesis.

### 4.5. Adipocyte Differentiation

Cells were induced to differentiate into adipocytes with an adipogenic-induction medium (AIM) consisting of DMEM supplemented with 9% horse serum, 450 µM 1-methyl-3-isobutylxanthine (IBMX), 250 nM dexamethasone, and 5 µg/mL insulin (Sigma-Aldrich, Hamburg, Germany) and 1 µM rosiglitazone (BRL 49653, Cayman Chemical, Ann Arbor, MI, USA). The medium was changed every 2–3 days.

### 4.6. Alkaline Phosphatase (ALP) Activity Assay

Cells were induced with OIM in a 96 well plate. ALP activity was determined by incubating the cells with 1 mg/mL of P-nitro phenyl phosphate in 50 mM NAHCO_3_ and 1 mM MgCl_2_ buffer (pH 9.6) at 37 °C for 20 min. Absorbance was measured at 405 nm. Cell viability was determined using the CellTiter-Blue^®^ cell viability assay according to the manufacturer’s instruction. The value of ALP activity was normalization to the value of cell viability and represented as a fold change over the control. Each sample was measured in 6 biological replicates.

### 4.7. Cytochemical Staining

#### 4.7.1. Alkaline Phosphatase Staining

Osteogenic cells were fixed with an acetone/citrate buffer pH 4.2 (1.5:1) for 5 min at room temperature. Cells were stained with Napthol-AS-TR-phosphate solution (Sigma-Aldrich, Hamburg, Germany) for 1 h at room temperature. The staining solution consists of 1:1 *v/v* Napthol-AS-TR-phosphate solution (Napthol-AS-TR-phosphate diluted 1:5 in H_2_O) and Fast Red TR solution (Sigma-Aldrich ApS, Hamburg, Germany) (diluted 1:1.2 in 0.1 M Tris buffer, pH 9.0).

#### 4.7.2. Alizarin Red S Staining and Quantification

Cells induced to osteogenic lineage were fixed with 70% ice-cold ethanol for 1 h at −20 °C and stained with Alizarin red (40 mM, pH = 4; Sigma-Aldrich, Hamburg, Germany) for 10 min at room temperature. To quantify calcium deposition, AR-S was eluted with 10% cetylpyridinium chloride (Sigma-Aldrich ApS, Hamburg, Germany) for 1 h at room temperature, and the absorbance was measured at 570 nm. Values were normalized to cell number and presented as a fold change over the control non-induced cells.

#### 4.7.3. Oil Red O Staining and Quantification

Differentiated cells into adipocytes were fixed in 4% paraformaldehyde for 10 min at room temperature. Accumulated fat droplets were stained with Oil Red O (0.5 g in 60% isopropanol) (Sigma-Aldrich, Hamburg, Germany) for 1 h. Oil Red O staining was eluted with isopropanol for 10 min at room temperature and lipids quantified from the extracted dye were measured at an absorbance of 490 nm. Oil Red O values were normalized to cell number (measured by number of viable cells) and then represented as a fold change over the control non-induced cells.

### 4.8. Western Blot Analysis

Cells were collected at specific time points in a lysis buffer (10 mM Tris-HCl, pH 7.4, 150 mM sodium chloride, 1% NP-40, 0.1% SDS, 1 mM EDTA, 1 mM phenyl-methylsulfonyl fluoride, 1 mM NaF, 1 mM Na_3_VO_4_), with a protease inhibitor cocktail (Roche Diagnostics, Mannheim, Germany). Then, 30 μg of protein was separated on 8–12% NuPAGE^®^ Novex^®^ Bis-Tris gel systems (Thermo Fisher Scientific, Dreieich, Germany), followed by transfer to a Hybond LFP polyvinylidene difluoride (PVDF) membrane (Millipore, Burlington, MA, USA). The membrane was blocked and incubated with a peroxidase-conjugated secondary antibody (Santa Cruz Biotechnology, Heidelberg Germany). Specific antibodies for total or phosphor p38 MAPK (Thr180/Tyr 182) and JNK (Thr183/Tyr185) were purchased from Cell Signaling Technology (Leiden, Netherlands). Phospho ERK1/2 (E4, sc-7383) and total ERK2 antibodies were purchased from Santa Cruz Biotechnology, Inc. Quantification of western blots was performed using an ImageJ program.

### 4.9. RNA Extraction and Real-Time PCR Analysis 

Total RNA was extracted from cells using a TRIzol single-step method (Thermo Fisher Scientific, Dreieich, Germany). Then, 1 µg of total RNA was used to synthesise cDNA with a revertAid H minus first strand cDNA synthesis kit (Fermentas, St Leon-Rot, Germany) according to the manufacturer’s instructions. Quantitative real time PCR (qPCR) was performed using an Applied Biosystems 7500 Real-Time system with Fast SYBR^®^ Green Master Mix (Applied Biosystems, city, CA, USA). Primer sequences for target genes were presented in additional file 1: Table S1. Target gene expression was normalization to β*-Actin* and *Hprt* mRNA expression as reference genes, using a comparative CT method [(1/(2delta-CT) formula, where delta-CT was the difference between CT-target and CT-reference] with Microsoft Excel 2007^®^ [65].

### 4.10. PCR Array Analysis

mBMSCs were induced to osteoblast differentiation in the presence or the absence of butein. Total RNA was extracted after 6 days of induction. A mouse osteogenic RT^2^ Profiler™ PCR array, containing 84 osteoblast-related genes (Qiagen Nordic, Sollentuna, Sweden) was performed using the SYBR^®^ Green qPCR method on an Applied Biosystems 7500 real-time PCR system. Upregulated genes by butein were represented as a fold change over the control (≥2 fold, *p* < 0.005) after normalization to the reference genes.

### 4.11. Statistical Analysis

All values were expressed as mean ± SD (standard deviation), of at least 3 independent experiments. Power calculation was performed for 2 samples using an unpaired Student’s T-test (2-tailed) assuming equal variation in the two groups. Differences were considered statistically significant at * *p* < 0.05, and ** *p* < 0.005.

## 5. Conclusions

Here, we identified butein as a new nutraceutical compound with a potential therapeutic effect for enhancing bone formation in bone-loss related diseases. Our data demonstrated the stimulatory effect of butein on the lineage commitment of BMSCs into osteoblasts on the process of adipocytes. The dual effects of butein for stimulating osteogenesis and inhibiting adipogenesis of BMSCs was found to be mediated via activating ERK1/2 phosphorylation. Thus, butein has a potential to be used as an osteo-anabolic drug for the treatment of osteoporotic patients.

## Figures and Tables

**Table 1:** List of upregulated osteogenic-related genes by butein in mBMSCs as measured by mouse osteogenesis RT^2^ Profiler™ PCR array analysis.

Gene Name	Gene Symbol	Fold Change
**Ossification and Matrix Molecules**		
Alkaline phosphatase, liver/bone/kidney	*Alpl*	6.2
Bone gamma carboxyglutamate protein	*Bglap*	5.8
Biglycan	*Bgn*	3.5
Collagen type I alpha 1	*Col1a1*	5.6
Collagen type I alpha 2	*Col1a2*	4.2
Collagen type V alpha 1	*Col5a1*	2.4
FMS-like tyrosine kinase 1	*Flt1*	3.6
Secreted phosphoprotein 1 (Osteopontin)	*Spp1*	4.3
**Cell Adhesion Molecules**		
Fibronectin 1	*Fn1*	2.1
Integrin beta 1 (fibronectin receptor beta)	*Itgb1*	2.3
Integrin alpha 2	*Itga2*	3.4
Integrin alpha 2b	*Itga2b*	2.2
Integrin alpha 3	*Itga3*	2.5
**Osteogenic Growth Factors**		
Fibroblast growth factor receptor 2	*Fgfr2*	3.6
Growth differentiation factor 10	*Gdf10*	2.8
Insulin-like growth factor 1	*Igf1*	3.4
Insulin-like growth factor I receptor	*Igf1r*	3.5
Platelet-derived growth factor alpha	*Pdgfa*	2.9
Vascular endothelial growth factor-A	*Vegf-a*	5.3
Vascular endothelial growth factor-B	*Vegf-b*	3.6
**Osteogenic Transcription Factors**		
Distal-less homeobox 5	*Dlx5*	4.6
Runt-related transcription factor 2	*Runx2*	3.4
Sp7 transcription factor 7	*Sp7*	5.2
Twist gene homolog 1	*Twist1*	3.7

## Supplementary Materials

Table S1: List of primers used for qPCR.
