# Supplementary material for: Butein Promotes Lineage Commitment of Bone Marrow-Derived Stem Cells into Osteoblasts via Modulating ERK1/2 Signaling Pathways
Source: Molecules. 2020 Apr 18;25(8):1885. doi: 10.3390/molecules25081885 (PMC7221720; doi:10.3390/molecules25081885)
Supplement: Supplementary file 1 [file molecules-25-01885-s001.pdf]

Supplementary table S1: List of primers used for qPCR

**Gene name**	**Forward primer 5'-3'**	**Reverse Primer 5'-3'**
β-Actin	GAT ATC GCT GCG CTG GTC GTC	ACG CAG CTC ATT GTA GAA GGT GTG G
Runx2	AGC AAC AGC AAC AAC AGC AG	GTA ATC TGA CTC TGT CCT TG
Ocn	CAG ACA AGT CCC ACA CAG CA	CTT TAT TTT GGA GCT GCT GT
Alp	GCC CTC TCC AAG ACA TAT A	CCA TGA TCA CGT CGA TAT CC
Osx	TATGCTCCGACCTCCTCAAC	AATAGGATTGGGCAGAAAG
Col1a1	GGT GAA CAG GGT GTT CCT GG	TTC GCA CCA GGT TGG CCA TC
Opn	GAA ACT CTT CCA AGC AAT TC	GGA CTA GCT TGT CCT TGT GG
Hprt	TCAGTCAACGGGGGACATAAA	GGGGCTGTACTGCTTAACCAG
Ppar-γ2	GGG TCA GCT CTT GTG AAT GG	CTG ATG CAC TGC CTA TGA GC
C/ebp-α	AAG CCA AGA AGT CGG TGG A	CAG TCC ACG GCT CAG CTG TTC
aP2	CAA AAT GTG TGA TGC CTT TGT G	CTC TTC CTT TGG CTC ATG CC
Lpl	CTGCTGGCGTAGCAGGAAGT	GCTGGAAAGTGCCTCCATTG
